# Spatial characterization of prostate cancer microenvironment with DRAQ5&Eosin fluorescence imaging and quantitative feature identification

**DOI:** 10.1016/j.jpi.2026.100746

**Published:** 2026-10-09

**Authors:** Michail Georgios Papachristos, Emiliano Spezi, Carolina Fuentes, Rachel J. Errington, Dimitris Parthimos

**Affiliations:** a School of Medicine, Division of Cancer and Genetics, Cardiff University, UHW Main Building, Heath Park, Cardiff CF14 4XN, UK; b Department of Biomedical Engineering & Biotechnology, Khalifa University, Shakhbout Bin Sultan St, Hadbat Al Za‘Faranah, Zone 1, Abu Dhabi, United Arab Emirates; c School of Computer Science and Informatics, Cardiff University, Abacws, Senghennydd Road, Cardiff CF24 4AG, UK

**Keywords:** Fluorescent microscopy, Prostate cancer, Machine learning, XGBoost, Pathomics, Gleason grading, DRAQ5, Eosin, Feature extraction

## Abstract

Prostate cancer progression is routinely quantified by expert pathologists on hematoxylin and eosin (H&E)-stained tissue using Gleason grading. However, tissue processing for H&E staining is very time-consuming, whereas Gleason grading is often subject to inter- and intraobserver variability. This diagnostic variability can be reduced through utilization of mathematically quantitative and standardized metrics to analyze the prostatic tissue microenvironment. In this study, we identify an optimal set of metrics that could potentially be used as a diagnostic biomarker signature, through a combination of tissue architectures and gradient boosting algorithms. We employed fluorescence microscopy imaged prostatic tissue, stained with DRAQ5&Eosin, which allows for rapid real-time staining and imaging of the tissue, as opposed to H&E, and also provides high-resolution nuclear and cytoplasmic images. We then used engineered algorithmic pipelines to extract intensity-based, morphological, and textural features from four different tissue components: epithelial nuclei, epithelial cytoplasm, stromal nuclei, and stromal cytoplasm. We developed classification models to distinguish healthy from cancerous prostatic regions based on the extracted features and identified the most predictive features. Our epithelial-based models achieved area under the curves (AUCs) in the range 0.646–0.896, whereas our stromal-based models attained AUCs in the range 0.742–0.840. Our DRAQ5&Eosin-based models were thus able to identify important tissue specific spatial biomarkers, allowing for the creation of nuclear and cytoplasmic spatial profiles for healthy and cancerous prostatic tissue respectively.

## Highlights

• Extracted handcrafted quantitative features from fluorescent DRAQ5&Eosin images of prostatic adenocarcinoma tissue.
• Spatially characterized prostate tumor microenvironment using gradient boosting algorithms.
• Identified and visualized important nuclear and cytoplasmic features associated with cancer progression from both epithelial tissue and surrounding microenvironment.

## Graphical abstract

**Figure:**
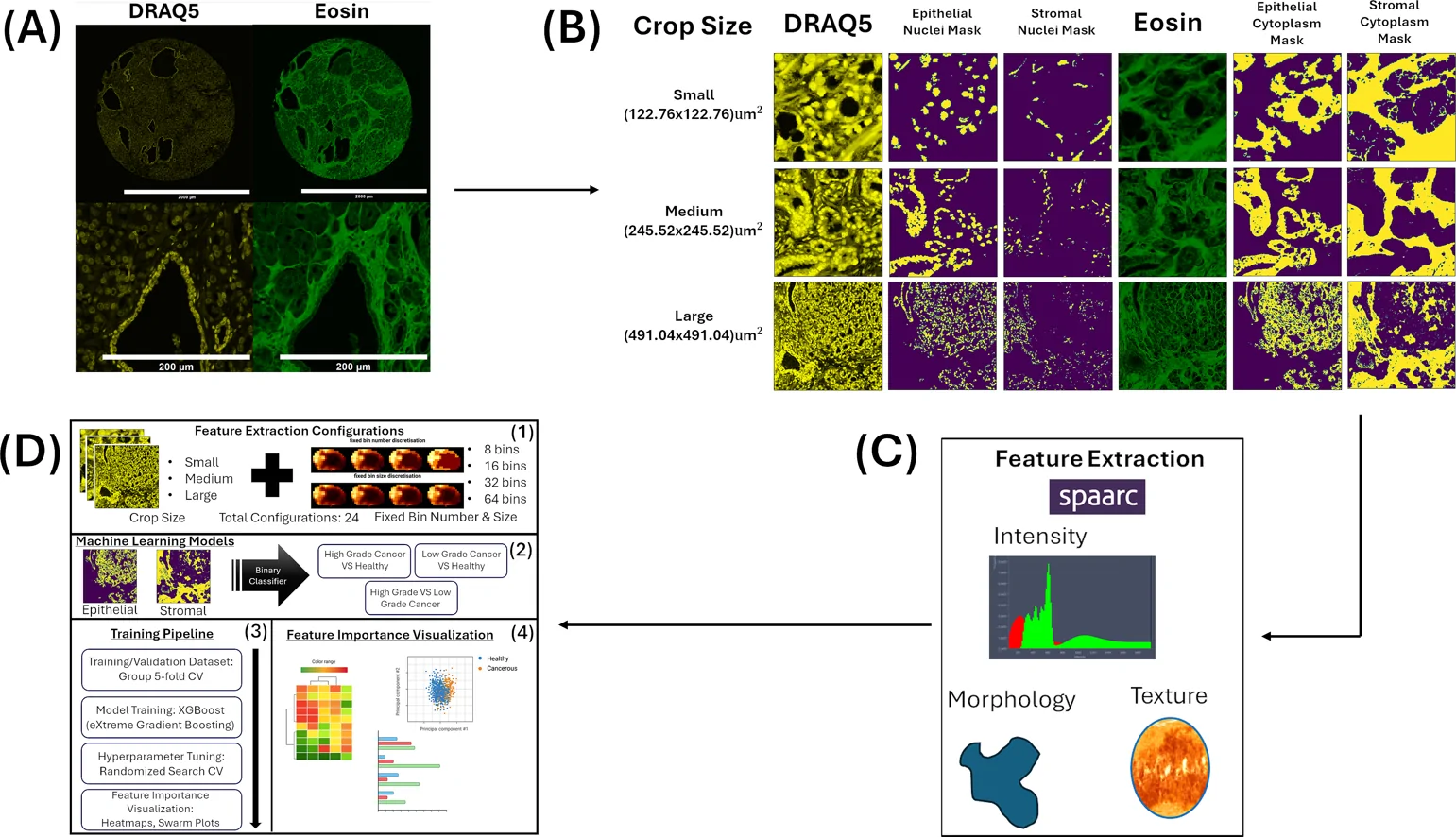

## Introduction

According to the Global Cancer Observatory by the World Health Organization, in 2022, there were 1.4 million new worldwide cases of prostate cancer, ranking as the fourth cancer type in terms of incidence, causing the death of almost 400,000 individuals.^1^ Prostatic adenocarcinoma incidence and progression is reflected in the tissue microstructure/microenvironment, which includes the epithelial gland and, importantly, its relationship and interaction with the surrounding stroma. This homeostatic and morphological disruption is manifested particularly during cancerous infiltration of the tissue.^2^ In order to identify and grade prostate cancer, expert pathologists visually observe the spatial architecture of prostatic tissue stained with hematoxylin and eosin (H&E), and assign a Gleason grade ranging from 1 to 5 at different epithelial regions.^3^ This standard grading system, however, is reliant on individual expertise and suffers from inter- and intraobserver variability.^4^ Utilizing mathematically quantifiable and standardized metrics to analyze the prostatic tissue microenvironment would lead to more robust and reproducible classification of distinct tissue patterns/architectures, and aid in more accurate clinical diagnosing. Characterizing how both the epithelial and stromal components of the tissue are affected is vital, considering that research in the role of stroma in prostate cancer is limited compared to other types of cancer.^2^

Over the last decade, there has been considerable advancement in the development of digital medical imaging. This involves digital pathology such as high-resolution whole-slide microscopy imaging,^5^ or digital radiology, focusing on radiological images such as computed tomography (CT), positron emission tomography (PET), or magnetic resonance imaging (MRI).^6^ Digital medical imaging allows for the potential incorporation of computational analysis and artificial intelligence in both research and clinical settings.^7^ Medical image analysis often incorporates deep learning and several other machine learning approaches.^8^ Whereas predictive, a major limitation of the use of deep learning is the lack of explainability and interpretability of models that are often considered black boxes. More explainable and interpretable methods of analysis include using handcrafted features for classification as opposed to the computational abstract extracted features of deep learning models. This mathematical quantification of different regions of interest is possible using feature extraction algorithms describing the intensity, morphology, and textural information, through the analytic framework known as radiomics^9^ or pathomics^10^ for digital radiology and digital pathology, respectively.

Most deep learning and pathomics approaches usually analyze the standard H&E-stained bright-field microscopy images. A more recent staining and imaging approach utilizes fluorescence microscopy (FM),^11^ which is able to provide high resolution quantitative images of the nuclear and cytoplasmic aspects of the tissue using nuclear fluorescent stains.^11^ An advantage of FM over H&E is the possibility of rapid real-time staining of the tissue,^12^ and annotation by pathologists utilizing synthetic H&E images generated from FM data.^13^ One method developed by Elfer et al. uses the DRAQ5 (highly-specific nuclear/DNA stain)^14^ and eosin fluorescent dyes to generate two different grayscale images, a DRAQ5 image containing the nuclear information of the tissue, and an eosin image containing the cytoplasmic information of the tissue.^15^

In the literature, there have been approaches utilizing pathomics to classify regions of prostatic tissue for different spatial patterns, including Gleason grading.^16^, ^17^ However, these studies used standard H&E staining for the pathomics analysis. To our knowledge, there is a research gap with regard to employing FM data instead, which can provide high spatial- and intensity-resolution multidimensional grayscale images, and thus can allow for specific biomarker identification of separate tissue characteristics through different stains. Identifying spatial biomarkers through pathomics can yield new insights into the biological significance of individual tissue components, as well as the interaction between the cancerous epithelium and its surrounding stromal microenvironment. Moreover, spatial profiling of tissue can potentially be used in the clinic for more precise characterization, prediction, and risk stratification of different types of prostatic adenocarcinoma.

In this work, we use the pathomics analysis feature extraction methodology on DRAQ5&Eosin fluorescent images to mathematically analyze and quantify how different regions of prostatic tissue alter from healthy to cancerous, and how they differentiate across Gleason grades and histological patterns. The objective of this explanatory study is to identify important epithelial and stromal tissue spatial features using machine learning algorithms and feature selection methods through the examination of both the nuclei and cytoplasm.

We hypothesize that: (a) Fluorescence-derived engineered pathomics can distinguish healthy vs cancer tissue, and high- vs low-grade cancer, similarly to existing H&E-based pathomics models. (b) Cancer-associated structural changes are more strongly expressed in epithelial than stromal compartments when quantified from DRAQ5&Eosin fluorescence images. (c) Morphological, intensity-based, and textural pathomics feature classes capture the known disorganization associated with prostate cancer progression.

## Methods and materials

### Overview of datasets

The training/validation dataset used in this study consists of two commercial tissue microarrays (TMAs) (“Commercial Tissue MicroArray A” and “Commercial Tissue MicroArray B”), and one additional TMA obtained from the Wales Cancer Biobank (WCB).^18^ The external testing dataset consists of 10 additional cores also provided from WCB.^18^ The Wales Cancer Biobank is approved as a Research Tissue Bank by Wales Research Ethics Committee 3 and is licensed by the Human Tissue Authority under the UK Human Tissue Act (2004) to store human tissue for research (research license 12107). It should be noted that there are no overlapping patients between the training/validation dataset and the external testing dataset, and that each of the 10 cores in the external testing dataset corresponds to a different patient. Additionally, the areas of the slides/cores in the external testing dataset are significantly larger than the cores originating from the TMAs of the training/validation, allowing for tissue heterogeneity within individual crops. A detailed schematic of the datasets is provided in Fig. 1.

**Fig. 1:**
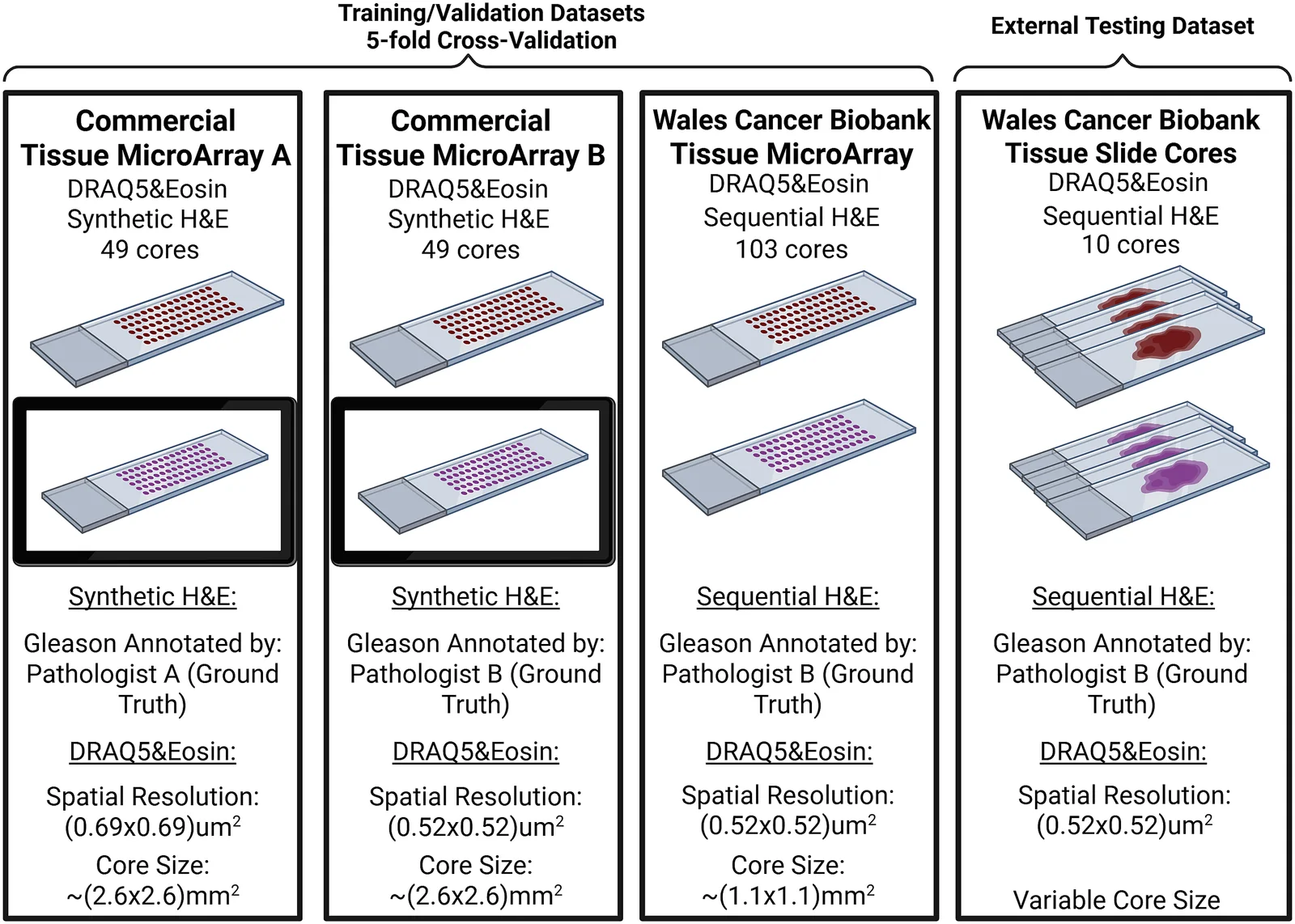
Schematic of datasets. The commercial TMAs consisted of 49 needle biopsy prostate cores (samples from the two TMAs are the same but the slices are not sequential). The WCB TMA consisted of 103 cores. Commercial TMAs A and B along with the WCB TMA were all used as training/validation datasets (with 5-fold cross-validation across the TMA cores). The WCB tissue slide cores were used as the external testing dataset. Figure adapted from Papachristos et al.^19^ (Creative Commons license: http://creativecommons.org/licenses/by/4.0/). Created in BioRender. https://biorender.com/bbcagwi.

These image datasets are the same used in Papachristos et al.,^19^ where the staining, imaging, data acquisition, and preprocessing protocols are outlined. The datasets contain for each core an unscaled 12-bit DRAQ5 and eosin fluorescent image, a Gleason pattern mask provided by an expert pathologist (Healthy, Gleason pattern 3, Gleason pattern 4, Cribriform pattern, Glomeruloid pattern, and Gleason pattern 5), and a coarse pseudo-generated epithelial mask. For the purposes of this study, nuclear and cytoplasmic masks were generated for the DRAQ5 and eosin images, respectively. The nuclear masks of the DRAQ5 images (rescaled to 0–4095) were created using Multi Otsu thresholding^20^ by the scikit-image package^21^ (number of bins set to 4096, classes set to 2). The cytoplasm masks of the eosin images (rescaled to 0–4095) were created using Multi Otsu thresholding^20^ by the scikit-image package^21^ (number of bins set to 4096, classes set to 3, with lower intensity threshold used to separate cytoplasm/background). Furthermore, the coarse epithelial masks were refined using a standard U-Net model trained on the training/validation dataset, and tuned on the external testing dataset, where the outputs of the U-Net model on both datasets were used as the refined epithelial masks (see Appendix A for more details regarding epithelial refinement).

The core images and masks were cropped into three different sizes: small (122.76 × 122.76) μm^2^, medium (245.52 × 245.52) μm^2^, and large (491.04 × 491.04) μm^2^. Before being cropped, each core image was contrast limited adaptive histogram equalized using the scikit-image package.^21^ The medium and large crops were resized to 256 ×256 pixels and the small crops were resized to 128 ×128 pixels using nearest-neighbor interpolation from the opencv python package.^22^ Each image crop also contains an associated refined epithelial mask, nuclei mask, cytoplasmic mask, Gleason pattern mask, most predominant pattern classification label, and patient of origin number.

Due to the size of the external testing dataset cores, there were some blurry crops on the edges of the tissue. The sharpness of each crop was quantified using the z-normalized variance of the Laplace filtered DRAQ5 image pixels within the crop's nuclei mask as a sharpness metric. Crops with a sharpness metric less than or equal to −1 were removed from the external testing dataset.

### Feature extraction

The Python programming language^23^ was used for all computational analysis through utilizing the Anaconda Navigator software^24^ and the JupyterLab/Notebook interface.^25^ The algorithms were run on the high-performance computing cluster Falcon from the Advanced Research Computing at Cardiff provided by the Wales Supercomputer infrastructure. Feature extraction was applied to the fluorescent DRAQ5&Eosin image crops. This was done using the Spaarc Pipeline for Automated Analysis and Radiomics Computing (SPAARC) (https://www.spaarc-radiomics.io).^26^, ^27^ SPAARC is a highly standardized package for the extraction of image features from both 2D and 3D medical images. It supports the extraction of 165 base features and 7 convolutional filters, all standardized following the guidelines of the Image Biomarker Standardization Initiative (https://theibsi.github.io).^9^, ^27^

From each DRAQ5 image crop, two masks were constructed: an epithelial nuclei mask, and a stromal nuclei mask. The epithelial nuclei mask was generated by intersecting the nuclei mask with the epithelial mask corresponding to the classified pattern, whereas the remaining nuclei (i.e., those outside epithelial regions) were used to construct the stromal nuclei mask. From each eosin image crop, an epithelial cytoplasm mask and a stromal cytoplasm mask were constructed similarly. A visualization of DRAQ5&Eosin crops along with their associated epithelial nuclei, epithelial cytoplasm, stromal nuclei, and stromal cytoplasm masks for the three different crop sizes are presented in Fig. 2. It should be noted that the stromal nuclear and cytoplasmic masks may include non-fibroblast related components, such as lymphocytes, endothelial cells, inflammatory cells, and segmentation artifacts. Thus, for the purposes of this study, stromal-based refers to regions involving the surrounding microenvironment as a whole.

**Fig. 2:**
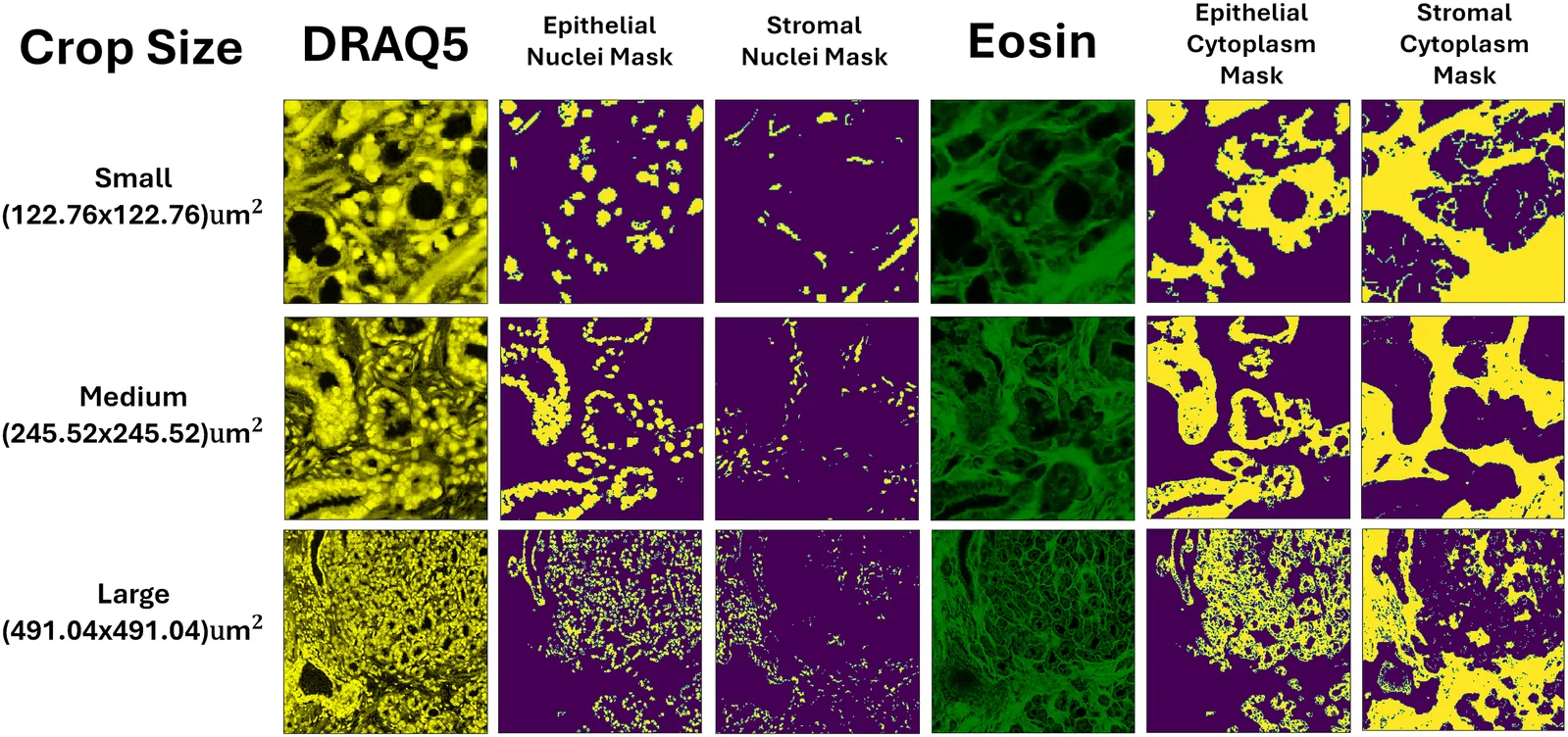
Visualization of grayscale DRAQ5 and eosin crops along with their associated masks for the three different crop sizes: small, medium, and large.

Features were extracted from the DRAQ5 crop using epithelial and stromal nuclei masks, and from the eosin crop using epithelial and stromal cytoplasm masks. The following feature categories were extracted: morphological, intensity-based statistics, intensity histograms, intensity-volume histogram, gray level co-occurrence matrix (GLCM), gray level run length matrix (GLRLM), gray level size zone matrix (GLSZM), gray level distance zone matrix (GLDZM), neighbourhood gray tone difference matrix (NGTDM), and neighbourhood gray level dependence matrix (NGLDM, coarseness parameter = 0) (812 total features). No interpolation or intensity range re-segmentation were used. The texture analysis type was set to 2D. Textural matrices and features (excluding GLSZM, and GLDZM) were extracted for three different distances. Short, medium, and long distances (1, 3, and 5) were used to capture texture information across fine, intermediate, and coarse spatial scales.

Because texture features can vary depending on how image intensities are discretized, we explored different intensity binning settings to identify the configuration that produced the most stable and informative features. We used the fixed bin number (FBN) and fixed bin size (FBS)^28^ methods with four bin values (8, 16, 32, and 64), creating eight possible feature-extraction configurations. Combined with the three crop sizes (small, medium, and large), this resulted in 12 total configurations for FBN intensity discretization models, and 12 total configurations for FBS intensity discretization models. The full machine learning pipeline described in the following section was applied to all configurations.

### Machine learning models

Two types of models were constructed for the analysis of the DRAQ5&Eosin crops: epithelial features-based and stromal features-based. For each type, we developed three binary classifiers: healthy vs low-grade cancer (Gleason 3), healthy vs high-grade cancer (Gleason 4 (including cribriform and glomeruloid) or Gleason 5), and low- vs high-grade cancer. Group 5-fold cross-validation was applied to the training/validation dataset using the StratifiedGroupKFold (for the healthy vs low grade, and low-grade cancer vs high-grade classifications) and GroupKFold (for the healthy vs high-grade classification) functions (scikit-learn package).^29^ The group used to split the folds was the patient of origin, meaning that no two folds contained crops originating from the same tissue sample, core, or patient. It should be mentioned that there was no patient overlap between the training/validation dataset, and the external testing dataset used to evaluate the developed models.

Within each training fold, the XGBoost^30^ machine learning algorithm was used to develop the binary classification model. For the determination of the optimal hyperparameters, a grid search method was used for the six different hyperparameters: learning rate (0.001, 0.01, and 0.1), max depth (3, 5, and 7), subsample (0.5, 0.75, and 1), column sample by tree (0.5, 0.75, and 1), number of estimators (100, 300, 500, and 700), and minimum sum of instance weight (hessian) needed in a child (3, 5, and 10). The optimal hyperparameters were determined using the RandomSearchCV function with 50 random iterations (scikit-learn package)^29^ with the cross-validation strategy being selected by the GroupShuffleSplit function. Patient of origin was used as group of split, number of splits was set to 3, and train size was set to 0.8. The objective of the XGBoost was set to logistic regression for binary classification, the evaluation metric was set to area under the curve (AUC), and the weights of classes were balanced to take into consideration class imbalance when training the models. Finally, the chosen models were assessed on the external testing dataset to measure their predictive power. To further examine in more detail how individual features contribute to the predictions of the models, the SHapley Additive exPlanations (SHAP)^31^ values of the external testing dataset were utilized, and visualized using Beeswarm plots.

This machine learning pipeline was applied to develop both model categories, each of which included three binary classification models for the analysis of DRAQ5&Eosin crops. Moreover, it was repeated for each of the eight feature extraction configurations, as well as three different crop sizes (small, medium, and large), resulting in a total of 12 total configurations for FBN intensity discretization models, and 12 total configurations for FBS intensity discretization models. A schematic of the machine learning pipeline is presented in Fig. 3.

**Fig. 3:**
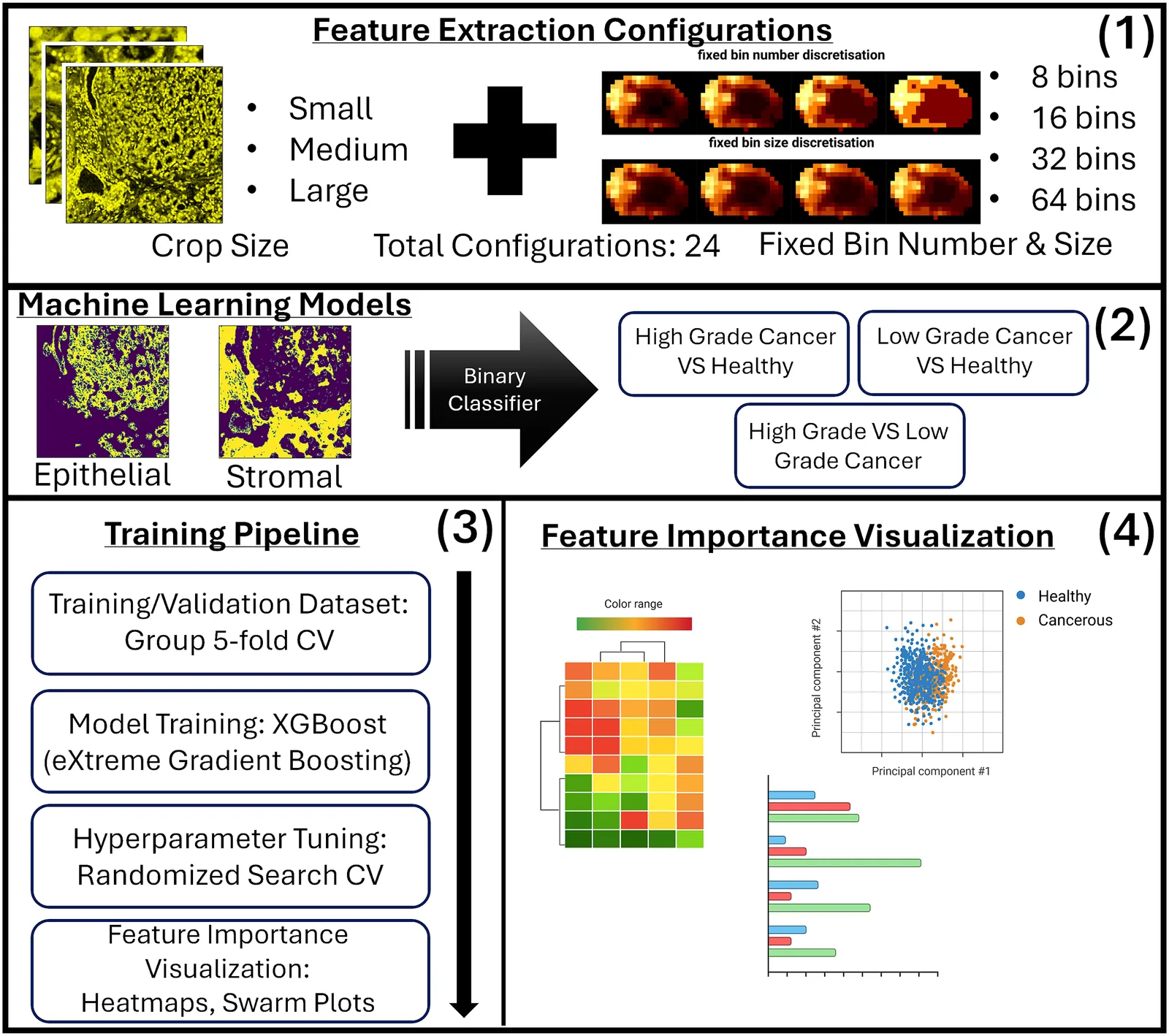
Schematic of machine learning pipeline. (1) The whole pipeline is run for all different possible configurations. Image adapted from Zwanenburg et al.^28^ (Creative Commons license: http://creativecommons.org/licenses/by/4.0/) (2) For each combination, two categories of models were constructed (epithelial- and stromal-based), and for each category, three binary classifiers were developed. (3) The training pipeline for the development of each model included Group 5-fold cross-validation, training using XGBoost, and hyperparameter tuning. (4) Feature importance visualization involved heatmaps of importance and Beeswarm plots (Icons created in BioRender. https://biorender.com/pvzj5hh).

## Results

### Optimal configurations

The optimal configurations for the models were obtained based on the training/validation dataset. The metric used for selecting the models was the F1-score averaged across the 5-folds used for training/validation. First, the optimal intensity discretization method was determined by comparing the average F1-score across all the epithelial- and stromal-based models. Table 1 contains the average macro-F1 and AUC scores across all the epithelial- and stromal-based models. We determined that FBN discretization yielded a higher training/validation average F1-score compared to FBS discretization for both model types. We further selected the optimal bin number and crop size for each model based on its individual training/validation F1-score across all the FBN models. For each configuration, the model with the highest macro-averaged F1-score across the 5-folds was chosen as the most predictive. In the following sections, we report results exclusively from the most predictive models. The optimal configurations across all the FBN models are presented in Table 2. The results for each individual configuration on the training/validation dataset can be found in Appendix B, Tables B.1, B.2, B.3, and B.4.

**Table 1:** Average macro-F1 and AUC scores across all the epithelial- and stromal-based models.

	Average training/Validation model performance				
	Epithelial-based			Stromal-based	
	Average F1-scores(%)			Average F1-scores(%)	
	FBN	FBS		FBN	FBS
Small	**69.70**	69.20	Small	**64.73**	64.37
Medium	72.03	**72.08**	Medium	**65.72**	64.68
Large	**75.22**	75.20	Large	**66.18**	64.94
Total	**72.32**	72.16	Total	**65.54**	64.67

	Average AUC scores			Average AUC scores	
	FBN	FBS		FBN	FBS

Small	**0.785**	0.781	Small	**0.734**	0.730
Medium	**0.815**	0.811	Medium	**0.745**	0.732
Large	0.851	**0.857**	Large	**0.783**	0.762
Total	**0.817**	0.816	Total	**0.754**	0.741

**Table 2:** Optimal bin number and crop size for each model selected based on its individual training/validation F1-score across all the FBN models.

	Optimal configurations			
	Epithelial-based		Stromal-based	
	Crop size	FBN	Crop size	FBN
Low grade vs Healthy	Large	16	Small	32
High grade vs Healthy	Large	64	Medium	8
High grade vs Low grade	Large	8	Large	8

### External testing data machine learning results

The training/validation dataset machine learning results can be found in Appendix C, Tables C.1, and C.2 and Figs. C.1, and C.2. To determine the predictive capability of the models developed, they were evaluated on an independent external testing dataset. Table 3 and Fig. 4 summarize the accuracies, macro-averaged F1-scores, AUCs, and ROC curves for all epithelial- and stromal-based models evaluated on the external test dataset.

**Table 3:** Crop-level aggregated (raw) accuracies, macro-averaged F1-scores, and AUCs of all the epithelial- and stromal-based models evaluated on the external testing dataset. 95% confidence intervals (calculated using bootstraping with 10,000 resamplings) are provided next to the metrics in square brackets.

	Crop-level results			
	Accuracy(%)	macro-F1(%)	AUC	Majority baseline (%)
Epithelial-based				
Low grade vs Healthy	62.19 [59.06, 65.21]	55.03 [51.79, 58.18]	0.646 [0.609, 0.683]	78.5
High grade vs Healthy	80.04 [76.41, 83.47]	78.54 [74.69, 82.25]	0.896 [0.867, 0.924]	58.47
High grade vs Low grade	72.13 [69.35, 74.81]	67.74 [64.65, 70.68]	0.759 [0.724, 0.794]	72.22
Stromal-based				
Low grade vs Healthy	74.05 [73.33, 74.75]	66.05 [65.20, 66.95]	0.777 [0.767, 0.786]	80.37
High grade vs Healthy	77.25 [75.44, 79.10]	76.87 [75, 78.64]	0.840 [0.823, 0.857]	59.23
High grade vs Low grade	70.98 [68.20, 73.75]	67.08 [64.01, 70.04]	0.742 [0.703, 0.780]	72.22

**Fig. 4:**
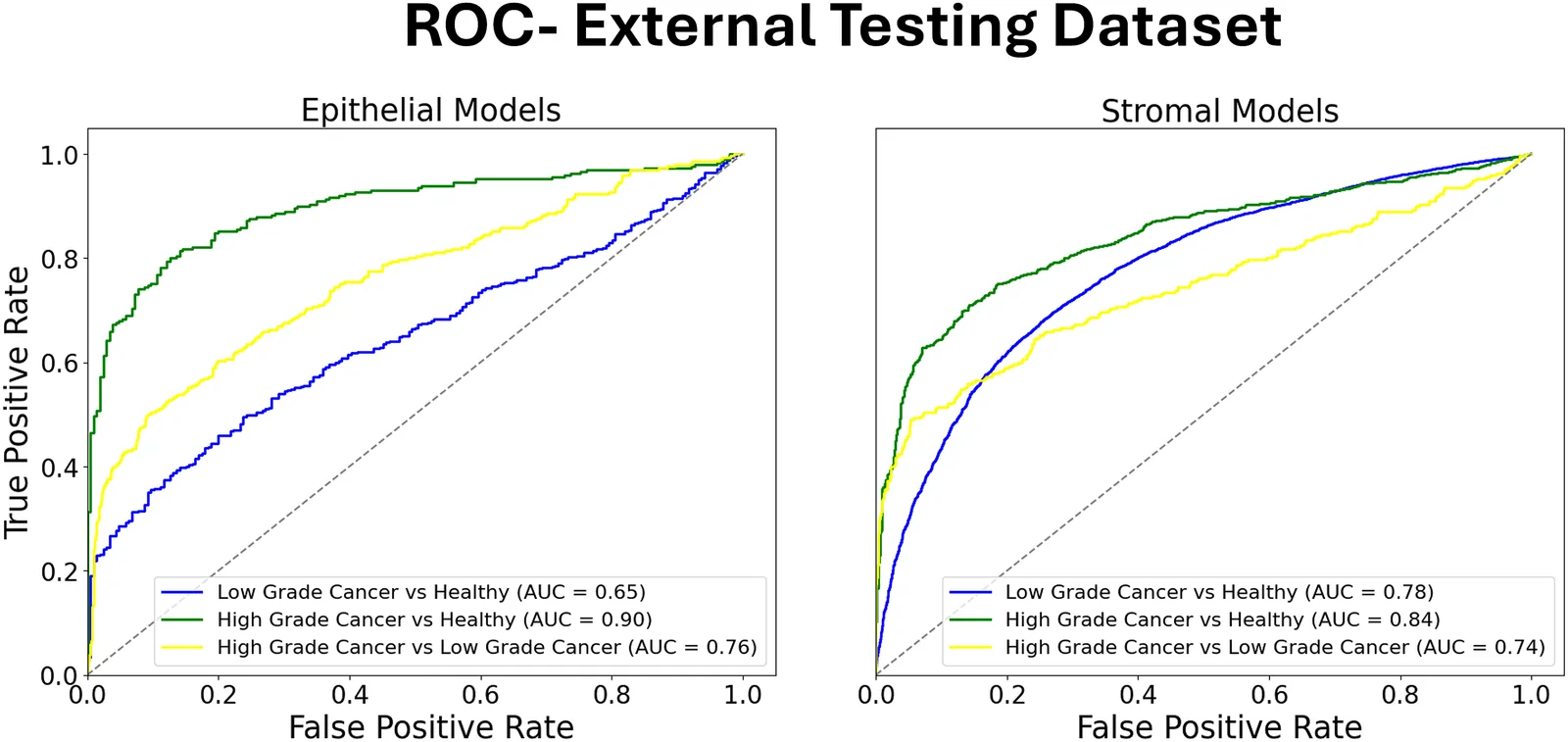
ROC curves of the models along with their associated AUCs on the external testing dataset: epithelial-based models (left), and stromal-based models (right).

For each model, performance was calculated at the core/patient level via majority voting across crops belonging to the same core. If a core contained no crops or less than 10 crops for a specific model, then that crop was not given a classification. Table 4 contains the patient-level aggregated accuracies for the 10 patients of all the epithelial- and stromal-based models, and the actual and predicted class for each core/patient. Fig. 5 contains the confusion matrices of the models on both crop- and patient-level.

**Table 4:** Patient-level aggregated (raw) accuracies for the 10 patients of all the epithelial- and stromal-based models evaluated on the external testing dataset. 95% confidence intervals (calculated using bootstraping with 10,000 resamplings) are provided next to the metrics in square brackets. Patient-level class is determined through majority voting with a minimum crop threshold of 10. The actual and predicted classes for each patient are given in parenthesis in the format of (actual/predicted class). H, healthy; G3, low grade; G45, high grade.

	Patient-level results		
	Accuracy(%)		
	Low grade vs Healthy	High grade vs Healthy	High grade vs Low grade
Epithelial-based			
Patient/Core 1	NA	98.31 [94.92, 100] (G45/G45)	96 [90, 100] (G45/G45)
Patient/Core 2	NA	94.12 [86.27, 100] (G45/G45)	80.39 [68.63, 90.20] (G45/G45)
Patient/Core 3	NA	90.91 [81.82, 98.18] (G45/G45)	23.64 [12.73, 34.55] (G45/G3)
Patient/Core 4	78.79 [63.64, 90.91] (G3/G3)	NA	87.88 [75.76, 96.97] (G3/G3)
Patient/Core 5	NA	57.50 [42.50, 72.50] (G45/G45)	0 [0,0] (G45/G3)
Patient/Core 6	64.91 [52.63, 77.19] (G3/G3)	NA	62.07 [50, 74.14] (G3/G3)
Patient/Core 7	81.82 [72.73, 90.91] (G3/G3)	NA	62.12 [50, 74.24] (G3/G3)
Patient/Core 8	NA	98.80 [96.39, 100] (G45/G45)	91.57 [85.54, 97.59] (G45/G45)
Patient/Core 9	48.09 [42.82, 53.37] (G3/H)	NA	79.77 [75.37, 83.87] (G3/G3)
Patient/Core 10	68.37 [64.14, 72.61] (G3/G3)	64.88 [58.05, 71.22] (H/H)	72.43 [66.91, 77.57] (G3/G3)
Stromal-based			
Patient/Core 1	82.64 [77.69, 87.19] (H/H)	84.68 [80.24, 89.11] (G45/G45)	88 [78, 96] (G45/G45)
Patient/Core 2	NA	61.62 [54.59, 68.65] (G45/G45)	84.31 [74.51, 94.12] (G45/G45)
Patient/Core 3	NA	96.12 [93.20, 98.54] (G45/G45)	27.28 [16.36, 40] (G45/G3)
Patient/Core 4	70.18 [66.89, 73.35] (G3/G3)	NA	39.39 [24.24, 57.58] (G3/G45)
Patient/Core 5	26.61 [19.35, 34.68] (H/G3)	57 [50, 64] (G45/G45)	22.86 [8.57, 37.14] (G45/G3)
Patient/Core 6	79.66 [76.75, 82.43] (G3/G3)	NA	79.31 [68.97, 89.66] (G3/G3)
Patient/Core 7	85.73 [83.53, 87.93] (G3/G3)	NA	87.88 [78.79, 95.45] (G3/G3)
Patient/Core 8	70.59 [47.06, 88.24] (G3/G3)	82.61 [78.55, 86.67] (G45/G45)	91.57 [85.54, 97.59] (G45/G45)
Patient/Core 9	71.46 [70.22, 72.65] (G3/G3)	55.88 [38.24, 73.53] (G45/H)	72.14 [67.45, 76.83] (G3/G3)
Patient/Core 10	74.89 [73.80, 75.94] (G3/G3)	77.89 [75.09, 80.68] (H/H)	70.59 [65.07, 76.10] (G3/G3)

**Fig. 5:**
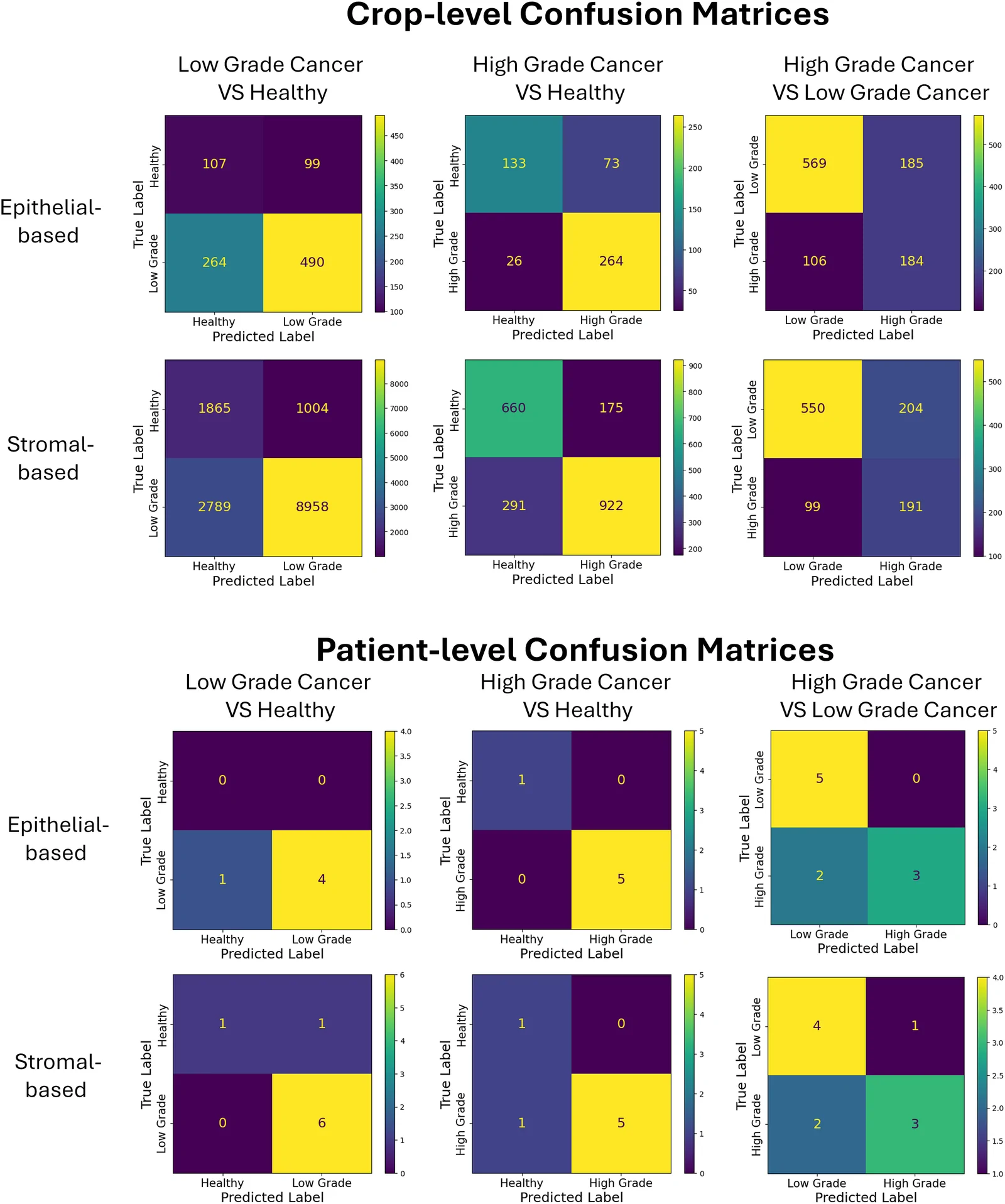
Crop- and patient-level confusion matrices of the models: low-grade cancer vs healthy (left), high-grade cancer vs healthy (center), and high- vs low-grade cancer (right) organized by epithelial-based models (top row), and stromal-based models (bottom row).

Finally, in order to gain greater model explainability, the impact of the features on the developed XGBoost outcomes are visualized. For each of the five cross-validation folds in a model, feature importance was determined using the mean of absolute SHAP values across the training samples. The features of each fold that make up 75% of the cumulative feature importance are considered significant. Common significant features across the 5-folds are defined as stable. In Fig. 6, Fig. 7, the SHAP Beeswarm plots of the 20 most impactful stable features of the external testing dataset for the epithelial- and stromal-based model outputs are presented, respectively. Non-stable features are not presented in the SHAP Beeswarm plot.

**Fig. 6:**
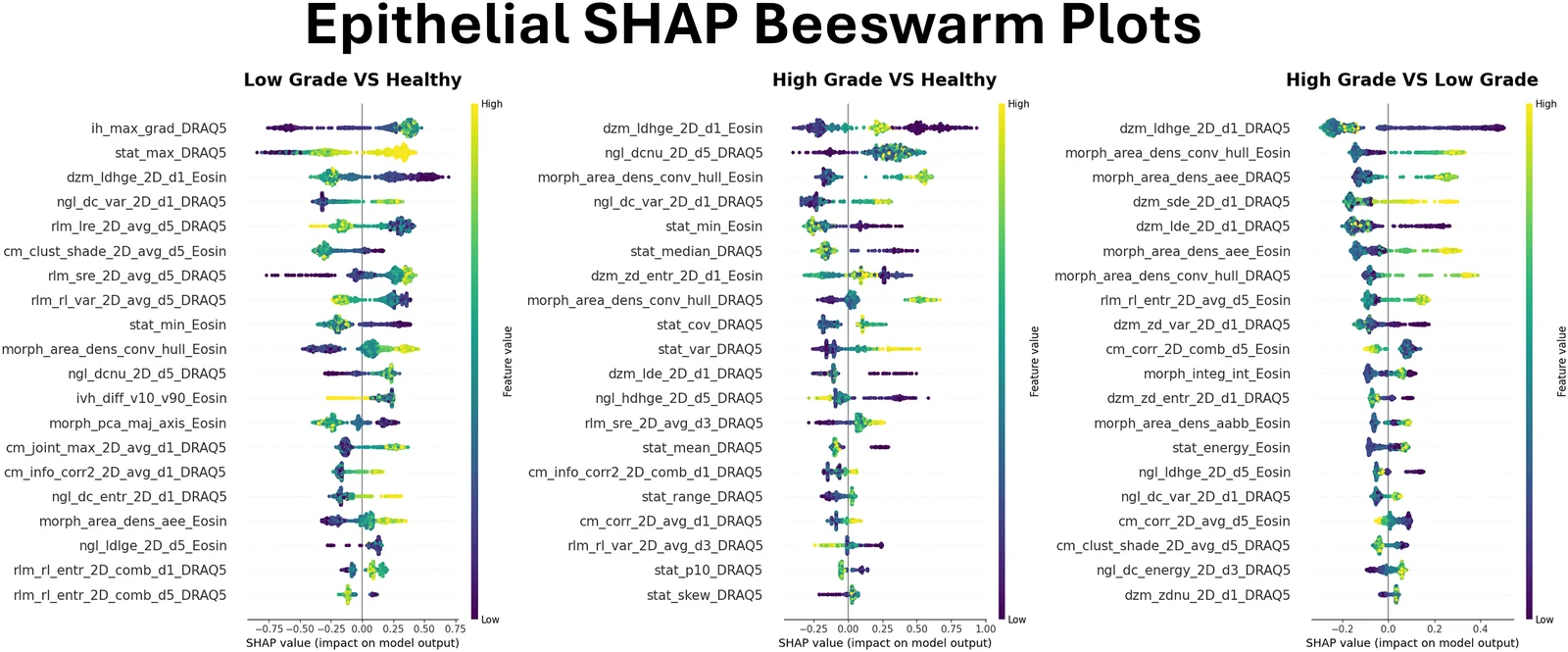
SHAP Beeswarm plots of 20 most impactful stable features (from top to bottom) of the external testing dataset for the epithelial-based models: low-grade cancer vs healthy (left), high-grade cancer vs healthy (center), and high- vs low-grade cancer (right).

**Fig. 7:**
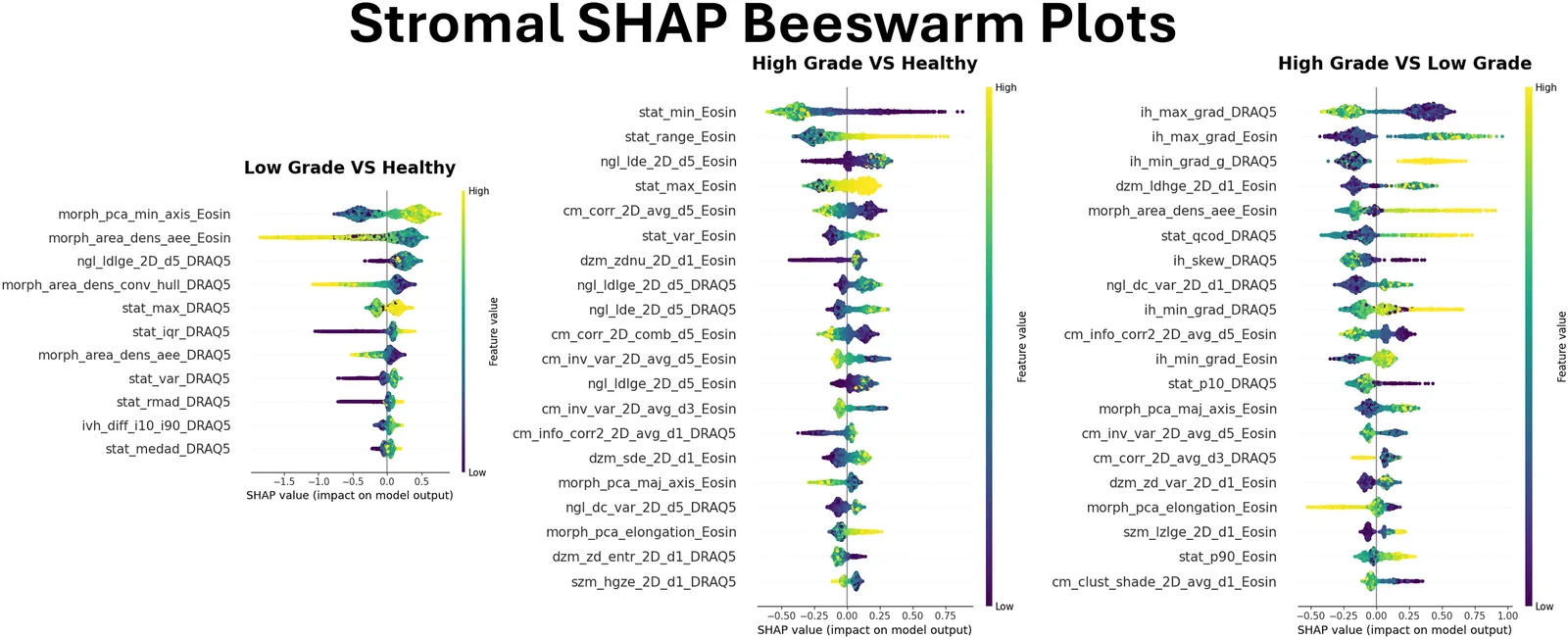
SHAP Beeswarm plots of 20 most impactful stable features (from top to bottom) of the external testing dataset for the stromal-based models: low-grade cancer vs healthy (left), high-grade cancer vs healthy (center), and high- vs low-grade cancer (right).

Further feature visualization can be found in Appendix D, Figs. D.1, D.2, D.3, D.4, and D.5. Details about the visualized features are explained in the Image Biomarker Standardization Initiative reference manual (https://arxiv.org/pdf/1612.07003).^9^, ^28^ The textural features contain distance metrics (d1, d3, and d5) where: (a) d1 captures fine textures and local intensity variations, but is sensitive to noise and small-scale heterogeneity, (b) d3 represents intermediate patterns, balancing local and regional texture and also reduces sensitivity to noise compared to the shorter distance, and (c) d5 captures coarse textures and global heterogeneity, and can be useful for detecting large-scale structural patterns. Overall, the feature codes are given here as such: (IBSI code)_(distance metric)_(image type) (GLSZM and GLDZM features do not contain distance metrics, so in the feature code, d1 is given as a default). The computational times required for feature extraction per configuration are reported in Appendix E, Table E.1. Information about data availability regarding the extracted features is reported in Appendix F.

## Discussion

Both models based on features extracted from the epithelium, and those based on features extracted from the surrounding stroma/microenvironment were found to be predictive when it comes to differentiating between healthy and cancerous tissue patterns. In terms of predictive power, the epithelial-based models were found to comparable to the stromal model with the exception of the low grade vs healthy classification, where the stromal-based models were more predictive. For the epithelial-based models, the most accurate was the classifier between high-grade cancer vs healthy tissue regions with an accuracy of 80.04% and an AUC of 0.896. Furthermore, regarding the stromal-based models, the differentiation between healthy and Gleason 3 regions achieved an accuracy of 74.05% with an AUC of 0.777, whereas the cancer stratification model (high- vs low-grade cancer) achieved a lower accuracy of 70.98% with an AUC of 0.742. In general, it seems that the models developed were able to differentiate between low- and high-grade cancer at a patient level, with epithelial-based models being able to classify all five low-grade cancer patients correctly, but misclassifying two out of the five high-grade patients as low-grade patients. The stromal-based models had the same results, with the exception of one low-grade patient that was classified as high-grade instead.

Overall, based on the SHAP values on the external testing dataset, the most influential DRAQ5 intensity features on the epithelial-based model outputs that were also positively correlated with cancer incidence or progression model prediction were: the maximum intensity, maximum gradient, coefficient of variation, variance, range, and skew. Negatively correlated DRAQ5 intensity features were: median, mean, and 10th percentile. For eosin, intensity-based energy was positively correlated, whereas minimum intensity and volume fraction difference between intensity fractions 10% and 90% were negatively correlated features. In the DRAQ5 stromal regions, the maximum intensity, interquartile range, variance, robust mean absolute deviation, median absolute deviation, volume fraction difference between intensity fractions 10% and 90%, minimum histogram gradient intensity, minimum gradient, and quartile coefficient of dispersion intensity features displayed positive correlations, whereas the maximum gradient, skew, and 10th percentile showed the opposite pattern. Finally, eosin stromal intensity features that had a positive correlation with SHAP values were maximum intensity, range, variance, maximum gradient, minimum gradient, and 90th percentile, whereas minimum intensity was negatively associated with the SHAP value.

Regarding the morphological characteristics, the morphological area density of both DRAQ5 and eosin were found to be consistently positively associated with cancer incidence or progression prediction across all epithelial-based models, with eosin major axis length displaying a decrease in the low grade vs healthy model. In the low grade vs healthy stromal-based model, morphological area density was found to be negatively associated for both DRAQ5 and eosin, whereas minor axis length was positively correlated with SHAP values. For the high grade vs healthy stromal-based model, morphological elongation showed a positive correlation with the SHAP values, whereas major axis length demonstrated the opposite. This pattern is reversed in the high- vs low-grade model, where morphological area density and major axis length has a positive model impact, whereas elongation has a negative one.

Additionally, the most important epithelial textural characteristics positively associated with cancer incidence or progression SHAP values were: NGLDM dependence count non-uniformity, NGLDM dependence count variance, GLRLM small runs emphasis, GLDZM small distance emphasis, and GLRLM run entropy. The most negatively associated epithelial textural features were: GLDZM large distance high gray-level emphasis, GLCM cluster shade, GLRLM run length variance, GLDZM zone distance entropy, NGLDM high dependence high gray-level emphasis, NGLDM large distance emphasis, GLDZM zone distance entropy. Finally, the SHAP positively associated stromal features were: NGLDM low dependence emphasis, NGLDM low dependence low gray-level emphasis, GLDZM zone distance non-uniformity, GLDZM low dependence high gray-level emphasis, and GLCM second measure of information theoretic correlation. The negatively correlated stromal textural features were: the GLCM correlation, GLCM inverse variance.

Related articles, such as Garcia et al.^16^ were able to achieve an AUC of 0.922 ±0.045 for distinguishing between benign and Gleason grade 3 glands using handcrafted features, whereas Brancato et al.^17^ attained an AUC of 0.97–0.99 for differentiating between non-malignant (including stroma) and malignant regions, and an AUC of 0.72–0.73 for separating Gleason 3 vs Gleason 4 or 5 regions. In work by Papachristos et al., deep learning models were used to analyze the same DRAQ5&Eosin data as our current feature-based models for prostate cancer tissue characterization.^19^ Comparable results were achieved, with AUCs of 0.9314, 0.8398, and 0.7715 for the high grade vs healthy, low grade vs healthy, and high- vs low-grade models, respectively.^19^ However, it should be mentioned that these articles make use of different methodologies and datasets, whereas also mainly focusing on predictability rather than tissue spatial profiling. Thus, whereas difficult to directly compare them with our current work, our results display an encouraging pattern. Moreover, a main advantage of the present models based on feature extraction is that they provide explainability because they rely on human-interpretable features. We were thus able to generate spatial profiles for different regions of healthy and cancerous tissue which is a major benefit over the developed deep learning models by Papachristos et al.^19^ This could potentially be clinically important, because fluorescent microscopy through nuclear markers allows for rapid real-time biopsy imaging.^11^, ^32^, ^33^

Cancer-associated structural changes in the DRAQ5&Eosin fluorescence images were overall found to be equally expressed in the stromal and epithelial components. The exception was the low grade vs healthy models, where the stromal-based model was more predictive. This demonstrates the potential importance of the surrounding stromal microenvironment, especially regarding initial prostate cancer development.

The tissue disorganization associated with prostate cancer progression was captured through intensity-based features in the epithelial nuclei (maximum gradient, coefficient of variation, quartile coefficient of dispersion, and robust mean absolute deviation), along with an increase in the DNA content of the nuclei (maximum intensity). Intensity variability also presented in a spatial level across the tissue, through increased textural characteristics such as NGLDM dependence count non-uniformity, NGLDM dependence count variance, NGLDM low dependence emphasis, and GLDZM small distance emphasis. This suggests that different characteristics of the spatial intensity distribution of the nuclei and cytoplasm seem to change not only from healthy to cancerous regions, but also especially between different grades of cancer, which is captured in both the epithelial tissue, but also in its surrounding stromal microenvironment. Biologically, these spatial local intensity and texture distribution alterations of the microenvironment could be linked to the tumor environment heterogeneity that is associated with prostate cancer.^34^ Spatial intensity non-uniformity in epithelial nuclei could be connected to cancerous prostatic biological processes such as chromatin dysregulation, remodeling, epigenetic modifications, 3D chromatin structure alteration, chromatin relaxation, and transcriptional plasticity,^35^ or epithelial–mesenchymal transition.^36^ Furthermore, stromal nuclei and cytoplasmic heterogeneity could be linked to microstructural cancerous changes, which occur in the reactive stroma, characterized by an abundance of myofibroblasts and carcinoma-associated fibroblasts, producing enzymes linked to extracellular matrix remodeling such as collagen type I, tenascin, and FAP.^37^, ^38^ Additionally, the identified stromal biomarkers could also be associated with non-stromal microenvironment-related biological processes, including angiogenesis due to release of vascular endothelial growth factor,^39^ and immune response of various different subtypes including tumor-infiltrating lymphocytes, tumor-associated macrophages, and tumor-specific antigens.^40^ Subsequent analysis could involve a combination of DRAQ5 and eosin staining with different types of immunohistochemical stains, to link the identified cancerous spatial profiles with more specific biomolecular processes.

Morphological spatial biomarkers were also identified, with the most prominent one being an increase in area density of both the epithelial nuclei and cytoplasm. This could potentially be because high Gleason grades are characterized by poorer gland differentiation, potentially presenting as less compact but more dense structures. Regarding the stroma, an initial decrease in cytoplasmic area density from healthy to Gleason 3 was pinpointed, followed by a subsequent increase from Gleason 3 to higher cancerous grades. Similarly to our study, Garcia et al.^16^ identified 136 relevant features for classification, where 94 of them were textural. Brancato et al.^17^ reported that the most important features for distinguishing high- vs low-grade cancer were wavelet transformation and local binary pattern features.

The main limitation of this study is the size of the datasets utilized, which affects the predictability of the models. The external test used for this study is derived from 10 tissue cores/patients, and thus the effective sample size of this study is 10 samples, which limits the statistical power. Furthermore, for the purpose of this kind of explanatory analysis and spatial profiling, it is important for the underlying masks used for feature extraction to be well defined. In our study, we used pseudo-generated epithelial masks that we refined using an U-Net model trained on the training/validation dataset, and tuned on the external testing dataset. This results in a well-defined separation between the epithelium/stroma, which is consistent across both datasets, but also potentially reduces the predictability of the models on foreign datasets, due to the epithelial masks on those datasets not necessarily being similarly defined, resulting in inconsistent feature extraction. In future studies, more stable mask annotations could be achieved through a combination of the DRAQ5 and eosin dyes with a fluorescent epithelial marker.

Another limitation is related to the inter-variability of the ground truth provided by the expert pathologists, which influences the training and validation of the proposed models, and potentially affects the robustness of the identified tissue biomarkers. From Papachristos et al.,^19^ an average quadratic weighted Cohen kappa score of 0.857 between Pathologist A and Pathologist B on a subset of nine small cores from the WCB TMA is reported. Future studies, which make use of bigger datasets and consensus-oriented annotation by multiple pathologists, could allow for a higher predictive power and statistical confidence of the important features identified. Additionally, a higher number of samples would allow to investigate features for better differentiation between Gleason 4 and Gleason 5, as well as specific architectural tissue patterns such as cribriform, glomeruloid, mucuous adenocarcinoma, and intraductal carcinoma.^41^

## Conclusion

This explanatory study demonstrates a computational approach for spatially profiling epithelial and stromal regions of the prostatic microenvironment using fluorescent nuclear and cytoplasmic markers. Furthermore it shows that these features can be linked to healthy, low-, and high-grade cancer phenotypes using feature extraction and gradient boosting algorithms. Interestingly, the stromal-based models were found to have a comparable, and in some cases, higher predictive power than their corresponding epithelial-based models, which highlights how crucial the relationship between stromal and epithelial tissue is in regards to cancer development and progression. The identified spatial biomarkers could establish a biologically meaningful link to known biological processes underlying poorly differentiated prostate cancer. Future replication and reproducibility studies using different datasets would strengthen the applicability of the identified spatial biomarkers.

## CRediT authorship contribution statement

1) Michail Georgios Papachristos (Conceptualization, Data curation, Formal analysis, Methodology, Software, Validation, Visualization, Writing – original draft), 2) Emiliano Spezi (Conceptualization, Funding acquisition, Methodology, Supervision, Writing – review & editing), 3) Carolina Fuentes (Conceptualization, Funding acquisition, Methodology, Supervision, Writing – review & editing), 4) Rachel J. Errington (Conceptualization, Funding acquisition, Investigation, Methodology, Resources, Supervision, Visualization, Writing – review & editing), and 5) Dimitris Parthimos (Conceptualization, Formal analysis, Funding acquisition, Investigation, Methodology, Project administration, Supervision, Validation, Writing – original draft, Writing – review & editing).

## Declaration of competing interest

The authors declare the following financial interests/personal relationships which may be considered as potential competing interests: Emiliano Spezi reports financial support was provided by Engineering and Physical Sciences Research Council (EPSRC). Rachel J Errington reports a relationship with BioStatus Ltd. that includes: equity or stocks. I, Rachel Errington, declare that I am a shareholder in BioStatus Ltd. The research presented in this article utilizes products manufactured by BioStatus Ltd. This affiliation has been disclosed in the interest of transparency and does not influence the integrity or objectivity of the scientific findings reported. If there are other authors, they declare that they have no known competing financial interests or personal relationships that could have appeared to influence the work reported in this article.

## Data Availability

The data and code used in this study can be found at: https://doi.org/10.5281/zenodo.22906542. The extracted features across all the configurations are stored in a GitHub repository at: https://github.com/mgpapachristos/DRAQ5_Eosin_Pathomics.

## Appendix A Supplementary data

Supplementary Material Appendices A-F.
