# Supplementary material for: Spatial characterization of prostate cancer microenvironment with DRAQ5&Eosin fluorescence imaging and quantitative feature identification
Source: J Pathol Inform. 2026 Oct 9;23:100746. doi: 10.1016/j.jpi.2026.100746 (PMC13763555; doi:10.1016/j.jpi.2026.100746)
Supplement: Supplementary file 1 [file mmc1.docx]

# Appendix A. Epithelial Segmentation Refinement

For more accurate segmentation and separation between the epithelial tis-sue and surrounding stromal tissue, the coarse ground-truth epithelial masks retrieved from the Papachristos et al. paper^1^ were refined. This was done through utilizing the developed cancer segmentation model from the Pa-pachristos et al. paper^1^ to yield an initial epithelial mask for each medium sized crop (pixels from model classified as healthy or cancerous) in both the training/validation and external testing datasets. Afterwards, an epithe-lial segmentation standard U-Net model^2^ (based on the cancer segmentation model outputs) for medium sized crops was developed consisting of 4 en-coders and 4 decoders, with 16, 32, 64, 128, 256, 128, 64, 32, and 16 number of filters respectively, and a sigmoid function as the activation function of the final convolutional layer. The loss function of the model used was binary cross-entropy, while the optimizer set was Adam with a learning rate of 0.01, and the number of epochs was set to 20. The U-Net model was trained on the medium sized crops of the training/validation dataset, and tuned on the crops of the external testing dataset. The outputs of the developed U-Net model (with a threshold of 0.5) on both dataset crops were used as the refined epithelial segmentation masks.

For epithelial mask refinement of the small and large sized crops, two more epithelial segmentation U-Net models with the same architecture and hyperparameters were developed. For training/tuning of the small sized crop U-Net model, each medium crop was split into four small crops. Regarding the large sized crop U-Net model, the Papachristos et al.^1^ cancer segmen-tation model was applied on each of the four corners of each large crop to yield a large sized initial coarse epithelial mask. Examples of refined ep-ithelial masks compared to ground-truth coarse epithelial masks for a small, medium, and large DRAQ5&Eosin crop of the external testing dataset are presented in Figure A.1.

Figure Appendix A.1: Examples of refined epithelial masks compared to ground-truth coarse epithelial masks for a small, medium, and large DRAQ5&Eosin crop of the external testing dataset.

.

# Appendix B. Configurations Table

Table B.1 reports the number of crops on the training/validation and ex-ternal testing datasets for different crop types and crop sizes. Table B.2 shows the number of patients and cores of all the epithelial-based and stromal-based models on both the training/validation and external testing datasets. Tables

B.3 and B.4 display the accuracies, macro-averaged F1 scores, and AUCs (averaged across the five folds) for all fixed bin number and fixed bin size discretization configurations respectively evaluated in the training/validation dataset.

| Crop Size | Crop Type | Training/Validation dataset | External Testing Dataset |
| --- | --- | --- | --- |
| Small | Healthy | 4738 | 2869 |
| Small | Low Grade | 6167 | 11747 |
| Small | High Grade | 5635 | 4597 |
| Medium | Healthy | 1282 | 835 |
| Medium | Low Grade | 1605 | 3100 |
| Medium | High Grade | 1469 | 1213 |
| Large | Healthy | 322 | 206 |
| Large | Low Grade | 409 | 754 |
| Large | High Grade | 370 | 290 |

Table Appendix B.1: Number of crops on the training/validation and external testing datasets for different crop types and crop sizes.

| Training/Validation Dataset | | |
| --- | --- | --- |
| Epithelial-based | | |
|  | Patients | Cores |
| Low Grade VS Healthy | 57 | 101 |
| High Grade VS Healthy | 56 | 102 |
| High Grade VS Low Grade | 63 | 112 |
| Stromal-based | | |
|  | Patients | Cores |
| Low Grade VS Healthy | 73 | 121 |
| High Grade VS Healthy | 69 | 117 |
| High Grade VS Low Grade | 63 | 112 |
|  |  |  |
| External Testing Dataset | | |
| Epithelial-based | | |
|  | Patients | Cores |
| Low Grade VS Healthy | 5 | 5 |
| High Grade VS Healthy | 6 | 6 |
| High Grade VS Low Grade | 10 | 10 |
| Stromal-based | | |
|  | Patients | Crops |
| Low Grade VS Healthy | 8 | 8 |
| High Grade VS Healthy | 7 | 7 |
| High Grade VS Low Grade | 10 | 10 |

Table Appendix B.2: Number of patients and cores of all the epithelial-based and stromal-based models on both the training/validation and external testing datasets.

Fixed Bin Number Discretization

Low Grade VS Healthy	High Grade VS Healthy	High Grade VS Low Grade

| Model Type | Crop Size | Bin Number | Acc(%) F1(%) AUC | | | Acc(%) F1(%) | | AUC | Acc(%) F1(%) AUC | | |
| --- | --- | --- | --- | --- | --- | --- | --- | --- | --- | --- | --- |
| Epithelial-based | Small | 8 | 75.99 | 73.84 | 0.834 | 78.98 | 77.22 | 0.874 | 60.52 | 57.73 | 0.632 |
| Epithelial-based | Small | 16 | 76.19 | 74.28 | 0.838 | 79.03 | 77.22 | 0.874 | 60.99 | 56.57 | 0.657 |
| Epithelial-based | Small | 32 | 75.03 | 73.00 | 0.820 | 78.81 | 77.12 | 0.872 | 63.28 | 60.55 | 0.673 |
| Epithelial-based | Small | 64 | 73.50 | 71.54 | 0.806 | 78.04 | 76.23 | 0.865 | 64.17 | 61.13 | 0.676 |
| Epithelial-based | Medium | 8 | 69.79 | 68.73 | 0.815 | 84.31 | 82.86 | 0.930 | 66.69 | 65.66 | 0.723 |
| Epithelial-based | Medium | 16 | 69.73 | 68.70 | 0.818 | 84.40 | 83.01 | 0.928 | 65.92 | 64.73 | 0.707 |
| Epithelial-based | Medium | 32 | 70.21 | 69.00 | 0.813 | 83.97 | 82.39 | 0.923 | 65.84 | 64.52 | 0.698 |
| Epithelial-based | Medium | 64 | 69.74 | 68.51 | 0.805 | 83.33 | 81.82 | 0.917 | 65.74 | 64.41 | 0.697 |
| Epithelial-based | Large | 8 | 73.89 | 72.25 | 0.822 | 83.42 | 82.74 | 0.926 | 67.51 | **66.25** | 0.785 |
| Epithelial-based | Large | 16 | 80.10 | **79.37** | 0.859 | 84.55 | 83.90 | 0.931 | 65.77 | 64.86 | 0.774 |
| Epithelial-based | Large | 32 | 79.20 | 77.66 | 0.869 | 85.02 | 84.26 | 0.933 | 66.41 | 65.21 | 0.765 |
| Epithelial-based | Large | 64 | 76.89 | 75.35 | 0.842 | 85.70 | **85.06** | 0.937 | 66.81 | 65.70 | 0.774 |
| Stomal-based | Small | 8 | 71.35 | 69.71 | 0.801 | 70.81 | 69.29 | 0.793 | 56.82 | 54.81 | 0.603 |
| Stomal-based | Small | 16 | 71.22 | 69.52 | 0.794 | 71.50 | 69.95 | 0.794 | 56.33 | 52.76 | 0.618 |
| Stomal-based | Small | 32 | 72.56 | **70.26** | 0.794 | 71.68 | 70.08 | 0.795 | 57.47 | 55.41 | 0.617 |
| Stomal-based | Small | 64 | 71.48 | 69.22 | 0.780 | 71.40 | 69.77 | 0.793 | 57.86 | 55.95 | 0.624 |
| Stomal-based | Medium | 8 | 64.49 | 63.07 | 0.735 | 74.13 | **72.39** | 0.835 | 62.10 | 60.79 | 0.658 |
| Stomal-based | Medium | 16 | 66.04 | 64.51 | 0.749 | 72.98 | 71.44 | 0.823 | 61.65 | 60.39 | 0.662 |
| Stomal-based | Medium | 32 | 65.56 | 63.98 | 0.750 | 73.74 | 72.20 | 0.822 | 63.47 | 62.19 | 0.679 |
| Stomal-based | Medium | 64 | 66.11 | 64.47 | 0.744 | 72.80 | 71.32 | 0.814 | 63.07 | 61.83 | 0.673 |
| Stomal-based | Large | 8 | 72.10 | 69.15 | 0.828 | 69.80 | 69.01 | 0.795 | 65.12 | **64.57** | 0.750 |
| Stomal-based | Large | 16 | 71.64 | 65.08 | 0.835 | 71.42 | 70.88 | 0.814 | 61.89 | 61.21 | 0.713 |
| Stomal-based | Large | 32 | 71.90 | 65.51 | 0.817 | 72.51 | 71.68 | 0.820 | 61.87 | 61.29 | 0.716 |
| Stomal-based | Large | 64 | 71.28 | 64.82 | 0.796 | 69.01 | 68.47 | 0.798 | 62.95 | 62.50 | 0.719 |
| Table Appendix B.3: | Raw accuracies (Acc), macro-averaged F1 scores, and AUCs (averaged across the five folds) | | | | | | | | |  |  |

for all fixed bin number discretization configurations evaluated in the training/validation dataset.

Fixed Bin Size Discretization

Low Grade VS Healthy	High Grade VS Healthy	High Grade VS Low Grade

| Model Type | Crop Size | Bin Number | Acc(%) F1(%) AUC | | | Acc(%) F1(%) | | AUC | Acc(%) F1(%) AUC | | |
| --- | --- | --- | --- | --- | --- | --- | --- | --- | --- | --- | --- |
| Epithelial-based | Small | 8 | 74.12 | 72.13 | 0.817 | 76.81 | 75.01 | 0.848 | 62.94 | 59.68 | 0.665 |
| Epithelial-based | Small | 16 | 74.75 | 72.75 | 0.819 | 77.10 | 75.26 | 0.858 | 61.83 | 58.83 | 0.655 |
| Epithelial-based | Small | 32 | 75.25 | 73.21 | 0.822 | 78.11 | 76.36 | 0.864 | 61.39 | 56.66 | 0.653 |
| Epithelial-based | Small | 64 | 75.66 | 73.76 | 0.834 | 78.60 | 76.81 | 0.868 | 63.07 | 59.90 | 0.665 |
| Epithelial-based | Medium | 8 | 68.98 | 67.80 | 0.795 | 83.64 | 81.90 | 0.913 | 65.89 | 64.49 | 0.703 |
| Epithelial-based | Medium | 16 | 70.61 | 69.37 | 0.815 | 83.56 | 81.96 | 0.917 | 65.26 | 63.87 | 0.706 |
| Epithelial-based | Medium | 32 | 71.30 | 70.02 | 0.815 | 84.20 | 82.56 | 0.919 | 65.23 | 64.26 | 0.698 |
| Epithelial-based | Medium | 64 | 73.29 | 72.44 | 0.830 | 84.14 | 82.64 | 0.925 | 64.98 | 63.69 | 0.699 |
| Epithelial-based | Large | 8 | 73.30 | 70.32 | 0.837 | 83.52 | 82.90 | 0.916 | 72.87 | 71.85 | 0.810 |
| Epithelial-based | Large | 16 | 72.19 | 69.52 | 0.823 | 82.31 | 81.67 | 0.908 | 71.61 | 70.54 | 0.806 |
| Epithelial-based | Large | 32 | 76.87 | 74.16 | 0.862 | 83.23 | 82.64 | 0.923 | 69.93 | 68.69 | 0.807 |
| Epithelial-based | Large | 64 | 78.81 | 77.41 | 0.867 | 83.73 | 83.09 | 0.927 | 70.53 | 69.57 | 0.801 |
| Stomal-based | Small | 8 | 71.87 | 69.61 | 0.790 | 70.23 | 68.68 | 0.783 | 56.85 | 54.87 | 0.614 |
| Stomal-based | Small | 16 | 72.31 | 69.99 | 0.794 | 70.89 | 69.24 | 0.788 | 56.39 | 54.49 | 0.606 |
| Stomal-based | Small | 32 | 71.55 | 69.41 | 0.785 | 70.71 | 69.13 | 0.786 | 56.34 | 52.59 | 0.607 |
| Stomal-based | Small | 64 | 72.17 | 69.84 | 0.796 | 71.61 | 69.98 | 0.797 | 56.38 | 54.68 | 0.610 |
| Stomal-based | Medium | 8 | 65.16 | 63.53 | 0.741 | 72.08 | 70.51 | 0.804 | 62.29 | 60.98 | 0.666 |
| Stomal-based | Medium | 16 | 64.65 | 62.99 | 0.740 | 71.23 | 69.70 | 0.800 | 62.02 | 60.70 | 0.664 |
| Stomal-based | Medium | 32 | 64.55 | 62.77 | 0.734 | 72.19 | 70.33 | 0.800 | 61.54 | 60.38 | 0.657 |
| Stomal-based | Medium | 64 | 66.50 | 65.10 | 0.737 | 74.17 | 72.31 | 0.815 | 58.11 | 56.89 | 0.627 |
| Stomal-based | Large | 8 | 69.44 | 62.88 | 0.756 | 69.88 | 69.17 | 0.783 | 64.16 | 63.57 | 0.731 |
| Stomal-based | Large | 16 | 71.10 | 64.42 | 0.815 | 70.62 | 69.82 | 0.782 | 64.32 | 63.70 | 0.728 |
| Stomal-based | Large | 32 | 70.72 | 64.15 | 0.768 | 69.28 | 68.56 | 0.780 | 61.61 | 61.21 | 0.723 |
| Stomal-based | Large | 64 | 70.17 | 63.76 | 0.779 | 67.40 | 66.72 | 0.778 | 61.86 | 61.31 | 0.720 |
| Table Appendix B.4: | Raw accuracies (Acc), macro-averaged F1 scores, and AUCs (averaged across the five folds) | | | | | | | | |  |  |

fixed bin size discretization configurations evaluated in the training/validation dataset.

# Appendix C. Training/Validation Data Machine Learning Results

The predictability of the models across the five folds of the training/validation data set are quantified in Table C.1 for the epithelial-based models and Table

C.2 for the stromal-based models. The ROC curves of the models across the five folds are visualized in Figure C.1. Figure C.2 contains the cumulative fea-ture importance for each fold of the trained models in the training/validation dataset.

Epithelial-based Models

Low Grade VS Healthy	High Grade VS Healthy	High Grade VS Low Grade Acc(%)	F1(%)	AUC	Acc(%)	F1(%)	AUC	Acc(%)	F1(%)	AUC

| Fold 1 77.05 75.35 0.891 79.47 78.98 | | | | | 0.918 | 72.04 71.49 0.772 | | |
| --- | --- | --- | --- | --- | --- | --- | --- | --- |
| Fold 2 | 64.77 62.85 | 0.666 | 89.26 | 89.24 | 0.962 | 67.57 | 64.67 | 0.743 |
| Fold 3 | 80.77 80.77 | 0.885 | 90.09 | 90.09 | 0.972 | 75.34 | 74.66 | 0.860 |
| Fold 4 | 100 100 | 1.000 | 83.09 | 81.40 | 0.902 | 86.09 | 85.85 | 0.919 |
| Fold 5 | 77.19 77.87 | 0.850 | 86.57 | 85.64 | 0.932 | 63.33 | 62.58 | 0.758 |

Table Appendix C.1: Raw accuracies (Acc), macro-averaged F1 scores, and AUCs of all epithelial-based models for each of the 5-folds.

Stromal-based Models

Low Grade VS Healthy	High Grade VS Healthy	High Grade VS Low Grade Acc(%)	F1(%)	AUC	Acc(%)	F1(%)	AUC	Acc(%)	F1(%)	AUC

| Fold 1 71.57 63.67 0.785 78.48 77.53 | 0.888 75.27 75.22 0.819 |
| --- | --- |
| Fold 2 73.33 72.86 0.800 67.10 65.90 | 0.793 61.08 59.46 0.641 |
| Fold 3 65.57 64.84 0.733 76.65 75.02 | 0.864 72.60 71.56 0.848 |
| Fold 4 82.96 80.90 0.899 68.91 65.42 | 0.752 69.57 69.57 0.771 |
| Fold 5 69.38 69.05 0.755 79.52 78.07 | 0.876 47.08 47.06 0.669 |

Table Appendix C.2: Raw accuracies (Acc), macro-averaged F1 scores, and AUCs of all stromal-based models for each of the 5-folds.

Figure Appendix C.1: ROC curves of the models along with their associated AUCs for each fold: low grade cancer vs healthy (left), high grade cancer vs healthy (center), and high grade cancer vs low grade cancer (right) organized by epithelial-based models (top row), and stromal-based models (bottom row).

.

Figure Appendix C.2: Cumulative feature importance for each fold of the trained models in the training/validation dataset: low grade cancer vs healthy (left), high grade cancer vs healthy (center), and high grade cancer vs low grade cancer (right) organized by epithelial-based models (top row), and stromal-based models (bottom row).

.

# Appendix D. Further Feature Visualization

From the trained XGBoost models, the importance of each feature was re-trieved. The weighted (averaged) feature importance (x100) of each DRAQ5 and Eosin feature category (Morphology, Intensity, Texture) was visualized through a heatmap plot generated using the Seaborn package. ^3^. In Figure D.1, the weighted importance (x100) per feature category is presented for all of the epithelial-based and stromal-based models respectively.

Figure Appendix D.1: Heatmaps of weighted importance (x100) per feature category for all of the epithelial-based and stromal-based models: low grade cancer vs healthy (left), high grade cancer vs healthy (center), and high grade cancer vs low grade cancer (right) organized by epithelial-based models (top row), and stromal-based models (bottom row).

.

To visualize how feature distributions differed between classes on the external testing dataset, we applied Principal Component Analysis (PCA) to the important features identified for each category after z-normalization. 4 The Silhouette Score was calculated (by keeping the number of PCs that had a cumulative variance less than 80%).^4^ For each Silhouette Score, a p-value was calculated through a permutation analysis where the labels were

shuffled 1000 times to develop a null distribution for comparison of statistical significance. A heatmap plot of the Silhouette score of each DRAQ5 and Eosin feature category was generated and is presented in Figure D.2. The first two principal components of each feature category are displayed as kernel density estimate (KDE) plots colored by class in Figure D.3.^3^

Figure Appendix D.2: Heatmap plot of the Silhouette score of each DRAQ5 and Eosin fea-ture category on the external testing dataset: low grade cancer vs healthy (left), high grade cancer vs healthy (center), and high grade cancer vs low grade cancer (right) organized by epithelial-based models (top row), and stromal-based models (bottom row).

Figure Appendix D.3: Kernel density estimate (KDE) plots of the first two principal components of each feature category colored by class on the external testing dataset: epithelial-based models (left), and stromal-based models (right) organized by low grade cancer vs healthy (top row), high grade cancer vs healthy (middle row), and high grade cancer vs low grade cancer classification (bottom row).

For each model, the mean of the absolute SHAP values of each feature was calculated, and then averaged by feature category. In Figure D.4 and

D.5, the average mean of absolute SHAP values of each feature category is plotted for the epithelial-based, and stromal-based models respectively.

Figure Appendix D.4: Barplot of the average mean of absolute SHAP values of each feature category of the external testing dataset for the epithelial-based models: low grade cancer vs healthy (left), high grade cancer vs healthy (center), and high grade cancer vs low grade cancer (right).

Figure Appendix D.5: Barplot of the average mean of absolute SHAP values of each feature category of the external testing dataset for the stromal-based models: low grade cancer vs healthy (left), high grade cancer vs healthy (center), and high grade cancer vs low grade cancer (right).

# Appendix E. Computational Time

The computational times required for feature extraction per configuration on the training/validation dataset and external testing dataset respectively are presented in Table E.1.

Feature Extraction Computational Time (minutes) Training/Validation Dataset	External Testing Dataset

| Crop Size | Bin Number | FBN | FBS | FBN | FBS |
| --- | --- | --- | --- | --- | --- |
| Small | 8 | 17 | 83 | 30 | 103 |
| Small | 16 | 21 | 44 | 41 | 52 |
| Small | 32 | 22 | 30 | 44 | 45 |
| Small | 64 | 34 | 31 | 56 | 31 |
| Medium | 8 | 15 | 36 | 18* | 56 |
| Medium | 16 | 16 | 26 | 19* | 35 |
| Medium | 32 | 20 | 19 | 24* | 22* |
| Medium | 64 | 24 | 18 | 28* | 28 |
| Large | 8 | 3 | 10 | 5 | 13 |
| Large | 16 | 4 | 9 | 6 | 10 |
| Large | 32 | 5 | 7 | 7 | 7 |
| Large | 64 | 6 | 5 | 8 | 7 |

Table Appendix E.1: Computational times (in minutes) required for feature extraction per configuration on the training/validation dataset and external testing dataset respectively. Values in asterisk (*) were not collected, and thus assumed based on the corresponding val-ues of the training/validation dataset (multiplication by samples ratio between datasets).

# Appendix F. Data Availability

The features extracted from the Training/Validation dataset and the Ex-ternal Testing dataset for each of the 24 configurations, along with the Python code used in this study can be found at [https://doi.org/10.5281/zenodo.](https://doi.org/10.5281/zenodo) 22906542.

# References

1. M. G. Papachristos, E. Spezi, C. Fuentes, I. Evangelou, D. H. Thomas,

F. Akinade, M. Wiltshire, A. Wilson, R. J. Errington, D. Parthimos, Prostate cancer tissue mapping and stratification using draq5 and eosin fluorescent labels integrated with ai classification and segmentation algo-rithms, PloS one 21 (3) (2026) e0345014.

2. O. Ronneberger, P. Fischer, T. Brox, U-net: Convolutional networks for biomedical image segmentation, in: International Conference on Medical image computing and computer-assisted intervention, Springer, 2015, pp. 234–241.
3. M. L. Waskom, seaborn: statistical data visualization, Journal of Open Source Software 6 (60) (2021) 3021. doi:10.21105/joss.03021.

URL <https://doi.org/10.21105/joss.03021>

4. F. Pedregosa, G. Varoquaux, A. Gramfort, V. Michel, B. Thirion,

O. Grisel, M. Blondel, P. Prettenhofer, R. Weiss, V. Dubourg, J. Vander-plas, A. Passos, D. Cournapeau, M. Brucher, M. Perrot, E. Duchesnay, Scikit-learn: Machine learning in Python, Journal of Machine Learning Research 12 (2011) 2825–2830.
